# Genetic Regulators and Physiological Significance of Glycogen Storage in *Candida albicans*

**DOI:** 10.3390/jof5040102

**Published:** 2019-10-30

**Authors:** Marcus A. Zeitz, Zainab Tanveer, Anatole T. Openshaw, Martin Schmidt

**Affiliations:** Department of Biochemistry and Nutrition, College of Osteopathic Medicine, Des Moines University, 3200 Grand Avenue, Des Moines, IA 50312, USA; Marcus.A.Zeitz@dmu.edu (M.A.Z.); z.tanveer@hotmail.com (Z.T.); Anatole.T.Openshaw@dmu.edu (A.T.O.)

**Keywords:** *Candida albicans*, glycogen, glycogen synthase *GSY1*, Efg1

## Abstract

The dimorphic human fungal pathogen *C. albicans* has broad metabolic flexibility that allows it to adapt to the nutrient conditions in different host habitats. *C. albicans* builds large carbohydrate stores (glycogen) at the end of exponential growth and begins consumption of stored carbohydrates when nutrients become limiting. The expression of genes required for the successful transition between host environments, including the factors controlling glycogen content, is controlled by protein kinase A signaling through the transcription factor Efg1. In addition to the inability to transition to hyphal growth, *C. albicans efg1* mutants have low glycogen content and reduced long-term survival, suggesting that carbohydrate storage is required for viability during prolonged culture. To test this assumption, we constructed a glycogen-deficient *C. albicans* mutant and assessed its viability during extended culture. Pathways and additional genetic factors controlling *C. albicans* glycogen synthesis were identified through the screening of mutant libraries for strains with low glycogen content. Finally, a part of the Efg1-regulon was screened for mutants with a shortened long-term survival phenotype. We found that glycogen deficiency does not affect long-term survival, growth, metabolic flexibility or morphology of *C. albicans*. We conclude that glycogen is not an important contributor to *C. albicans* fitness.

## 1. Introduction

Microbes need to carefully manage macronutrients such as carbohydrates to survive the challenges of supporting energy metabolism in an ever-changing environment. In yeast, the constant availability of carbohydrates is important for survival and growth: In this organism carbohydrates are essential for both aerobic and anaerobic energy metabolism as well as for the synthesis of a cell wall that contains a large amount of the carbohydrates glucose, mannose and chitin [1,2].

The fundamental mechanisms of yeast carbohydrate storage and their regulation have been established in the well-researched model yeast *Saccharomyces cerevisiae* [3]. This fungus synthesizes glycogen as the main form of carbohydrate storage when carbon, nitrogen or phosphorous become limiting [4]. Glycogen is a large polymer of α-1,4 linked glucose subunits that branch with α-1,6 glyosidic bonds, forming a 20 nm cytosolic particle with a molecular weight of 10^7^ to 10^8^ Da [5,6]. In *S. cerevisiae* glycogen reserves are mobilized during times of nutrient deprivation [7] and give cells a small fitness advantage in competitive growth assays [8]. However, ablation of glycogen synthesis activity in *S. cerevisiae* affects neither viability nor stress response, suggesting that in this yeast glycogen is not essential for growth and survival under laboratory conditions [9]. There are two, functionally different glycogen synthase genes in *S. cerevisiae* [9], *GSY1* and *GSY2*, both of which are controlled by the protein kinase A signaling pathway through the transcription factors Msn2 and Msn4 (reviewed by [10]). Deletion of both Msn2 and Msn4 reduces cellular glycogen content, but also has more far-reaching implications on homeostasis that leave the cell hypersensitive to a variety of environmental stresses [11,12], notably carbon source starvation stress [13]. 

The pathogenic yeast *Candida albicans* controls the expression of glycogen synthase similarly to *S. cerevisiae*. In *C. albicans*, the expression of the sole putative glycogen synthase gene, *GSY1*, is controlled by the *Candida*-specific transcription factor Efg1 [14], whose activity responds to input from the protein kinase A (PKA) signaling cascade [15]. In addition to glycogen synthesis, Efg1 controls many other important aspects of *C. albicans* biology on the transcriptional level, from the transition to filamentous growth to the balance between cytoplasmic glycolysis and mitochondrial respiration [14,16]. *C. albicans* homozygous *efg1* mutants have reduced virulence, presumably because of the importance of Efg1 for the adaptations required to survive within its host [17]. 

The present study was undertaken to establish the role of glycogen in maintaining *C. albicans* viability during long-term culture, to identify genetic factors regulating glycogen synthesis and to determine if the reduced virulence of an *efg1* null mutant can be explained by the low glycogen content of the strain. 

## 2. Materials and Methods 

### 2.1. Strains and Culture Conditions

*C. albicans* strains used in this study are listed in Table 1. 

Cells were grown at 30 °C in yeast/peptone/dextrose (YPD) media (1% *w*/*v* yeast extract, 2 % *w*/*v* peptone, 2% *w*/*v* agar added as needed; Thermo Fisher, Walton, MA). The pH of media containing 2% acetate or lactate instead of glucose was adjusted to 6.5. To induce the null mutant genotype in the Gene Replacement and Conditional Expression (GRACE) collection mutants, YPD medium was supplemented with 100 µg/mL tetracycline.

### 2.2. Assessing Survival

Survival of cells on solid surfaces was assessed by either a serial dilution or by a mechanical isolation test. For both assays, overnight YPD cultures of yeast were harvested, washed with sterile water, plated on solid media/surfaces at 4 × 10^8^ cells per plate and incubated at 30 °C. At the indicated times, cells were scraped from the agar surface for assessment of survival. For the serial dilution test, cells were suspended in sterile PBS, counted in a hematocytometer, serially diluted, plated on fresh YPD plates and incubated at 30 °C for 2 days. Viability was determined by dividing the number of colony-forming units by the number of cells plated. Time points were measured in triplicate and the experiment was repeated three times. For mechanical isolation, approximately 2 × 10^6^ cells were scraped off the plates with a toothpick at the indicated times, streaked on fresh media and incubated as described above. To assess survival on polystyrene, 10 µL drops of water containing 2 × 10^6^ cells were applied to the surface of a Petri dish and incubated at 30 °C. At the indicated times cells were recovered by suspending in sterile water. Time points were measured in duplicate and the experiment was repeated three times. 

Loss of viability was scored by examining the streaking pattern for growth of colonies, with total absence of growth corresponding to a survival of below 5 × 10^−7^ viable cells/total cells. The mechanical isolation method was validated by comparison with dilution assays performed on the same set of plates. Survival was assessed on YP media (containing 2% glucose, lactose or acetate) as well as on dry polystyrene surfaces without any nutrient supplementation. We found that strain survival differed remarkably depending on the growth substrate, the genetic background of the mutants and at times even during experimental repeats. To standardize the experiment, we scored a mutant as having poor long-term survival if no growth occurred in the streak assay 20 % earlier than in the corresponding WT under the same conditions (a difference of 4 days, in most cases); experiments were repeated at least three times. Both methods for assessment of survival were found to be feasible only for *C. albicans* strains with yeast or pseudohyphal morphologies; mutants with constitutive hyphal morphology or excessive biofilm formation do not have mechanically separable colony-forming units and cannot be assessed with either protocol. 

### 2.3. Strain Construction

Glycogen synthase *gsy1* null mutants were constructed using the short-flanking homology method [23]. Deletion fragments were amplified by PCR from plasmids pGEM-HIS1 and pRS-ARGΔSpe1 using primers GSY1-Del-Up ATACAATTTCTGTAATTTTTCCGATAAAAATAGTAATACCACCAGATGAATTGATTATCCAAGACTCACTAATTTTGGGTGTTTTCCCAGTCACGACGTT and GSY1-Del-Down AAAATACAATTGAAACTTTGTAAGAAACCTATACATATGTATATAATTTATAAACAAGCATATAATATTGGGCAAAAATTTGTGGAATTGTGAGCGGATA. Integration of *HIS1* and *ARG4* reporter genes into the *GSY1* locus of strain SN152 was confirmed by PCR and by sequencing the genomic locus of the integration site. To rule out second-site suppressor effects, three independent isolates of *gsy1/gsy1* strains were obtained and tested in parallel. 

### 2.4. Determination of Glycogen Content―Iodine Method

The *C. albicans* mutant collections screened in this study were supplied as frozen stocks in 96-well microtiter plates. To normalize the starting titer for growth experiments, cells were transferred after thawing into 200 µL of fresh YPD medium with a 96-pin replicator and grown to saturation for 2 days at 30 °C. From these cultures, cells were spotted onto fresh YPD medium in Omnitray Plates (NUNC, Roskilde, Denmark), grown for 16 h at 30 °C, exposed to iodine vapor and photographed when color development was deemed satisfactory (10–30 min). 

### 2.5. Determination of Glycogen Content―Enzymatic Method

Cellular glycogen content was analyzed with an enzymatic method modified from Wang et al. [24]. Yeast cells were harvested from liquid cultures or transferred from solid media into PBS, collected by centrifugation and flash frozen in liquid nitrogen. Pellets were thawed in 0.2 mL 20% KOH, boiled for 1 h, chilled on ice and neutralized with 0.15 mL of 5 M HCl. Glycogen was precipitated by adding 0.7 mL of ethanol and incubating at −20 °C for 30 min. Precipitate was collected by centrifugation (10,000 g, 5 min) and washed with 66% ethanol. Pellets were dried and suspended in 0.4 mL 50 mM sodium acetate, 5 mM CaCl2, pH 4.8. Glycogen was digested with 10 U of α-amylase and 1 U amyloglycosidase for 16 h at 37 °C. Detection of glucose released from glycogen pellets was performed in 100 µL assays containing 250 mM triethanolamine hydrochloride, 2.5 mM MgSO4, 125 mM ATP, 10 mM NADP, 0.5 U glucose 6 phosphate dehydrogenase, 0.5 U hexokinase and 10 µL of sample. NADPH production was quantified by correlating OD_340nm_ after 30 min incubation at RT with values obtained with a glycogen standard solution prepared from oyster glycogen (Sigma-Aldrich, St. Louis, MO). Glycogen content of cell pellets was normalized to dry cell weight: an aliquot of the culture used to determine glycogen was filtered through a glass fiber filter (Pall A/E) and the dry weight of the retentate was determined after drying for 2 h at 60 °C. Two cultures were sampled, and glycogen in each culture sample was measured in duplicate. Significance was determined with paired, two-tailed student *T*-tests.

### 2.6. Determination of Lipid Content―Nile Red Stain

The neutral lipid content of cells was estimated with a Nile Red staining protocol (adapted from [25]). Cells were collected by centrifugation (30 s 500 × g), washed with PBS and suspended in the same buffer. Nile Red dye was added from a DMSO stock solution to a final concentration of 5 µg/mL. After 5 min incubation in the dark, cell fluorescence in the long wavelength channel FL-2 was recorded with a FacScan flow cytometer (Becton Dickinson, Franklin Lakes, NJ). Fluorescence of mutants was normalized to wildtype fluorescence to obtain Fluorescence Increase Over Control values (FIOC). Three experiments with three technical repeats each were analyzed.

## 3. Results

### 3.1. Glycogen Deposits Decay More Rapidly Than Cell Viability During Long-Term Culture

**Figure 1:**
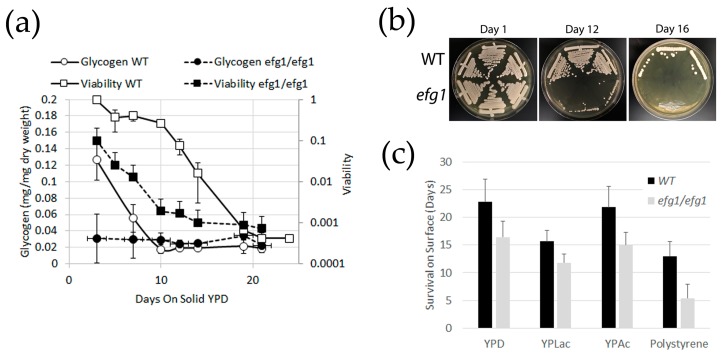
Glycogen content and viability of *C. albicans* wildtype (WT) SN152 and corresponding *efg1/efg1* mutant. (**a**) Glycogen content of WT reaches a low after 10 days of culture on YPD media. Viability declines around day 10 of culture. *efg1* null mutants have a low glycogen content from the start and viability declines more rapidly than in the WT; (**b**) Validation of a mechanical isolation assay to assess viability of cultures, confirming loss of viability at day 16 (WT) and 12 (*efg1*/*efg1*). (**c**) Viability of *efg1/efg1* mutant on a variety of solid media. In each case, the difference between WT and mutant is significant (*p* < 0.05).

Glycogen content and survival were analyzed in cultures of the WT SN152 and *efg1/efg1* mutant strains from the Transcriptional Regulator Knockout collection [19] grown at 30 °C on solid YPD medium. *efg1* mutants were included in this assay for two reasons. First, because of the incidental finding that the mutant had poor long-term survival and second, because of the expected finding that the mutant had low glycogen content (the expression of the *GSY1* gene is under the control of the Efg1 regulator [14]). As expected, viability and glycogen content decline over time: While glycogen in the WT strain is completely spent after 10 days, cultures retain residual viability for several days further (we interpret the detection of very small amounts of glycogen in the later stages of the experiment as a complete lack of glycogen, as the enzymatic assay is not entirely specific and detects small amounts of glycogen even in cells without glycogen synthase activity [9]). The same characteristic is true for *efg1/efg1* mutants, with the notable observation that in this strain both glycogen content and survival time are reduced compared to the WT (Figure 1a). To validate the simplified procedure for assessment of survival, a mechanical isolation test was performed on the cultures subjected to the serial dilution colony count assay. Both tests showed similar results, showing a loss of viability after days 16 and 12 for WT and *efg1/efg1* mutants, respectively (Figure 1b). The shortened survival of *efg1/efg1* mutants was confirmed in two strain backgrounds (Cai4-based HLC52 [17] and SN152-derivative from the Transcriptional Regulator Knockout collection [19]); it was further determined that the shortened survival phenotype of *efg1/efg1* mutants was not dependent on glucose as the carbon source, as a similar disadvantage was observed on non-fermentable carbon sources (YP medium containing 2% lactate or acetate instead of glucose), as well as on dry polystyrene surface without any growth substrate (Figure 1c). Efg1-deficieny had the most dramatic influence on survival on dry polystyrene surfaces, with the *efg1/efg1* mutants showing a decline in viability starting after just one day of exposure and a complete loss of viability on day 4. Strain survival in liquid YPD medium at 30 °C was assessed with the mechanical separation assay. The data show that cells survive in liquid media for much longer than on solid media, with a significantly reduced survival time for the *efg1/efg1* mutant (46 days for WT, 30 days for *efg1/efg1* from Transcriptional Regulator Knockout collection). Note that while survival time varied significantly between experiments, the difference between WT and *efg1/efg1* mutants was evident each time; t-testing for significance was thus performed using paired samples and yielded *p* values below 5% for every condition shown. 

### 3.2. Construction and Characterization of a Glycogen Synthase 1 Mutant

**Figure 2:**
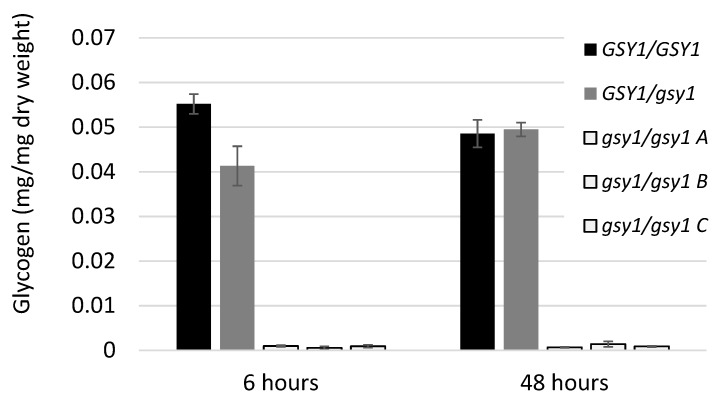
Glycogen content of WT, heterozygous and homozygous glycogen synthase mutants in exponential (6 h) and stationary (48 h) phase of growth. Glycogen content of three independent *gsy1/gsy1* isolates is shown. The difference in glycogen content between WT and *gsy1/gsy1* mutant is significant at the 5% level.

The CR_00780C_A reading frame is annotated as *GSY1*, the sole *C. albicans* glycogen synthase gene in *C. albicans* (Candida Genome Database, http://www.candidagenome.org/ [26]). Substitutions of the *GSY1* reading frame and 1000 bp of promoter sequence with *ARG4* and *HIS1* markers were obtained with the short-flanking homology method and verified by sequencing. While the heterozygous *gsy1::ARG4/GSY1* strain CaMS626 had near-WT glycogen content, the homozygous *gsy1::ARG4/gsy1::HIS1* strains CaMS627A,B and C had only 1.5 % and 2.0 % of the WT glycogen content during exponential and stationary phase of growth, respectively (Figure 2). Based on the aforementioned inherent limitation of the detection method, we interpret the low glycogen value in *gsy1/gsy1* mutants as absence of glycogen.

The homozygous *gsy1* null mutant strain CaMS627 showed no growth retardation in rich media, no grossly abnormal content of neutral lipids (as measured by Nile Red staining), no morphological phenotype and only a slight delay in germ tube induction at 37 °C in YPD with 10% fetal bovine serum. Furthermore, the *gsy1/gsy1* mutant had normal survival on solid surfaces on all tested media as well as normal resistance to the glyoxylate cycle inhibitors itaconic acid and perillic alcohol [27,28].

### 3.3. Identification of Genetic Factors for Glycogen Synthesis through Mutant Screening

To identify genetic factors and metabolic pathways involved in the regulation of *C. albicans* glycogen synthesis, three collections of homozygous deletion mutants containing 3165 strains were screened with the iodine vapor method for low glycogen content. The screen assessed 2853 separate deletions (46% of the *C. albicans* genome), as some ORF deletions were present in more than one collection. Reproducibility of data was achieved by confirming that strain duplicates in the Homan et al. [19] and Noble et al. [20] collections showed the same phenotype. The GRACE method of generating conditional null mutants does not favor the emergence of secondary site suppressors and thus does not contain duplicate strains [18]; in this case, the enzymatic determination of glycogen content was conducted with three independently induced clones of the same strain.

Of the 71 strains found to have low glycogen content by iodine vapor stain, 25 were confirmed to have significantly lower glycogen content in enzymatic assays. Table 2 lists the strains that have passed the following screening criteria: 1.) All duplicates of the ORF deletion showed weak glycogen staining with iodine vapor when compared to the corresponding WT and 2.) All duplicates were significant for low glycogen at the 5% level in repeated enzymatic glycogen determinations. A Gene Ontology [29] analysis of the 25 strains that met both criteria against the background of the 2853 screened mutants showed that the processes of: 1.) generation of precursor metabolites and energy, 2.) aerobic respiration and 3.) cellular respiration were significantly overrepresented in the low-glycogen group of strains.

### 3.4. Role of Efg1 Antagonists Tup1/Nrg1 in the Control of Glycogen Content 

**Figure 3:**
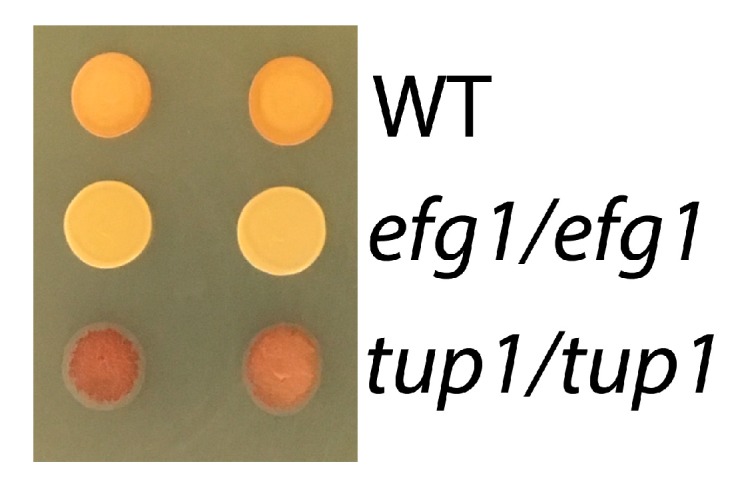
Glycogen content of *efg1/efg1* and *tup1/tup1* mutants from Homan et al. library assessed with the iodine vapor method. Ten microliters of a stationary culture were grown for 6 h on YPD plates and exposed to iodine vapor for 15 min (in duplicate). The intensity of brown coloration is a measure of glycogen content (*efg1/efg1:* low; *tup1/tup1:* high).

To further elucidate the contribution of the Efg1 regulon to glycogen content and long-term survival, strains deficient in the Efg1 antagonists Tup1 and Nrg1 were assessed with the methods describe above. It was found that the *tup1/tup1* (from Homan et al. collection) and *nrg1/nrg1* mutants (from both the Noble et al. and Homan et al. collections) had significantly elevated glycogen content. Although the *tup1/tup1* mutant could not be assessed with the enzymatic glycogen protocol because of its slow and morphologically aberrant growth, it showed high glycogen content when examined with the iodine vapor method (Figure 3) and the *nrg1/nrg1* mutant showed a glycogen content of 0.073 *+/−* 0.023 mg/mg (151% of WT). Viability of *nrg1/nrg1* and *tup1/tup1* mutants could not reliably assessed because of the strains’ constitutive hyphal morphology.

### 3.5. Efg1 Target Genes And Low-Glycogen Strains Have No Long-Term Survival Deficit

To follow up on the chance discovery that the glycogen-poor *efg1/efg1* mutants of *C. albicans* lose viability quickly on solid media, we utilized the previously validated mechanical isolation test to investigate the correlation of glycogen content and long-term survival. It was found that none of the 25 low-glycogen mutants identified in the screening showed significantly reduced survival time on solid media. To specify the role of Efg1-mediated gene regulation in maintaining viability, we retrieved 65 strains with homozygous mutations in known Efg1 target genes [14] from the libraries and examined their long-term survival on YPD media with the mechanical isolation assay. None of the mutants met the criteria for a survival defect.

## 4. Discussion

### 4.1. Glycogen in Yeast 

Glycogen is a macromolecule that is widely used for the intracellular storage of carbohydrates—not just in higher eukaryotes, but also in bacteria and yeast [3]. In the model yeast S. cerevisiae, glycogen deposits rise at the end of exponential growth and decline as cells progress through stationary phase [30]. With the size of glycogen deposits reaching 5–10 % of dry cell weight, one would assume that these massive carbohydrate stores fulfill an essential role in the yeast starvation response. However, *S. cerevisiae gsy1/gsy2* mutants completely devoid of glycogen synthase activity do not have a noticeable survival deficit [9] and mild phenotypic consequences of the deficiency become obvious only during competitive fitness assays [8]. 

Much less is known about glycogen synthesis and mobilization in the dimorphic yeast C. albicans, even though this organism is of major importance for human health: *C. albicans* is the leading cause for mycosis-associated mortality in the United States and poses a serious problem for health systems due to its ability to colonize immunocompromised patients in the hospital environment [31]. *C. albicans* is a versatile yeast that colonizes different habitats within the human host. The fungus lives in the yeast form as a commensal on the skin and mucous membranes. Under the influence of environmental host factors such as elevated temperature, CO_2_ and the presence of serum components the fungus undergoes dramatic changes in metabolism, membrane transport, morphology and colony organization that increase its ability to survive and spread within the host [32,33]. During colonization of the host, *C. albicans* needs to adapt to the availability of different external energy sources as well as balance the utilization of cellular lipid and carbohydrate stores for the generation of metabolic energy [34,35,36,37,38,39]. It has been recognized that survival within the host requires *C. albicans* to have significant metabolic flexibility. And indeed, inhibitors of energy redistribution between lipid and carbohydrate metabolism—glyoxylate cycle inhibitors—have shown potential as antifungal agents [27,28]. 

The transcription factor Efg1 is central to the above described adaptions of *C. albicans* to the host environment. The Efg1 regulon is composed of at least 283 target genes that control morphology, stress resistance and metabolism [14] and, as a consequence, *efg1/efg1* mutants show adaptation defects that greatly reduce virulence [17,40]. Among the Efg1 target genes are glycogen synthase and its antagonist, the glycogen degrading enzyme glycogen phosphorylase (*GSY1* and *GPH1*, respectively), implicating that stimulation of glycogen metabolism is part of the Efg1-mediated metabolic adaptations to life within the human host [41].

### 4.2. Glycogen and Viability of C. albicans efg1 Mutants in Long-term Culture 

The study was prompted by the chance observation that *C. albicans efg1/efg1* mutants have a very low glycogen content and do not survive extended periods of starvation well. Our findings related to mutant glycogen content show that transcriptional regulation by Efg1 increases glycogen deposition, just as the Efg1-counterregualtors Tup1/Nrg1 keep the glycogen content from increasing beyond normal levels. These findings suggest on one hand that the induction of glycogen synthase *GSY1* by Efg1 has a stronger metabolic effect than the parallel induction of the glycogen phosphorylase *GPH1*. On the other hand, it also suggests that the concomitant regulation of glycolytic and oxidative phosphorylation genes by Efg1 [14] creates a metabolic situation that favors the deposition of glycogen through regulation of glycogen synthase/phosphorylase enzyme activity (see review of mechanisms by [3]).

The role of Efg1 in supporting the transcriptional adaptation to the environmental stresses within the human host goes beyond the regulation of metabolism and carbohydrate storage. It includes the initiation of the yeast-to-hyphal transition, changing the composition of the cell wall and induction of radical stress defense mechanisms [14]. While the loss of any one of these functions could explain the observed deficit in long-term survival on solid surfaces, we chose to focus this study on the role of energy storage, hypothesizing that glycogen synthesis is important to maintain viability during starvation.

### 4.3. Glycogen is Consumed During Starvation, But is not Essential for Survival 

Our data show that *C. albicans* SN152 glycogen reserves are consumed during the first ten days of culture on YPD media and that loss of viability accelerates as glycogen reserves are spent. While this correlation of carbohydrate reserves and viability suggests that both factors are linked, the following experiments failed to show the necessity of glycogen for long-term starvation, or for that matter, any of the examined physiological processes. We found that a homozygous deletion of the *GSY1* reading frame on the R chromosome produced a strain devoid of glycogen that had no defects in growth, survival or morphogenesis. As is the case for *S. cerevisiae* [9], glycogen deficiency in *C. albicans* appears to have no obvious negative consequences under laboratory conditions. Hopes that glycogen synthesis could be a target for the development of novel antifungal agents are thus probably misplaced. 

This poses an interesting question: If carbohydrate reserves in the form of glycogen are not required to maintain viability during extended periods of starvation, where does the metabolic energy come from instead? It is conceivable that mobilization of lipid reserves can provide enough metabolic precursors to support cellular functions. In contrast to the situation in human organs, yeast commands the ability to produce carbohydrates from fatty acids through the glyoxylate cycle and thus enjoys a metabolic flexibility that improves its chances of survival in the host and other hostile environments [35]. However, we found no evidence that fatty acid metabolism and the glyoxylate cycle compensate for the loss of carbohydrate stores in glycogen deficient mutants. Neither do *gsy1/gsy1* mutants have any gross abnormalities in lipid content (by Nile Red staining), nor are they sensitive to glyoxylate cycle inhibitors (agents which prevent the conversion of stored fatty acids to carbohydrates). This leaves autophagy as the most likely source of metabolic energy during starvation in glycogen-deficient yeast: During starvation, yeast increase vacuolar degradation of cytoplasmic molecules, mostly protein, to meet cellular nutrient demand. The findings that defects in the autophagocytic pathway decrease starvation survival in *C. albicans* [42,43] suggest that autophagy makes a major contribution to homeostasis during long-term culture. We speculate that an increase in autophagy can compensate for the loss of stored carbohydrate energy in glycogen-deficient yeast. 

### 4.4. Genetic Factors Supporting Glycogen Synthesis in C. albicans 

A screening of three *C. albicans* mutant libraries identified 25 strains―fewer than 1 % of the screened clones―with significantly reduced glycogen content. Even though the screened strain collections were not comprehensive (covering 46 % of the genome) and had a certain bias (enriched for transcription factors and potentially essential genes), we were able to ascertain that genes in the gene ontology (GO) process domains of “generation of precursor metabolites/energy” and “respiration” make a strong contribution to the low glycogen phenotype. The findings from this experiment are hardly surprising, given the importance of energy homeostasis for carbohydrate storage―but they nevertheless demonstrate that low glycogen content is a rare phenotype that is specific to mutants with disturbances in carbohydrate energy metabolism.

### 4.5. The Role of Efg1 in Glycogen Storage and Long-Term Survival

Our data clearly show that Efg1 has roles in 1.) conserving viability during long-term culture on solid surfaces and 2.) in the maintenance of glycogen deposits. However, the data also show that these two functions are not linked as we were able to demonstrate that glycogen content is not correlated with survival. We conclude that members of the Efg1 regulon other than glycogen synthase must be responsible for preventing an early loss of viability during long-term culture. Our efforts to identify these factors through a screening of 65 strains with mutations in known Efg1 target genes failed to yield insights as no single life-shortening mutation could be identified. While this might be explained by the limitations of the available mutant pools (only 23 % of Efg1 targets screened, bias towards essential genes), it could also mean that there is no single life-sustaining factor. The survival benefits of Efg1 could be due to a synergistic action of all the stress responses initiated by this versatile transcription factor. This model of the role of Efg1 in *C. albicans mirrors* the situation in *S. cerevisiae*, where the transcription factors Msn2/Msn4 coordinate the expression of a large number of stress response genes, including the expression of the glycogen synthases [12,13] that do not measurably contribute to stress resistance [9]. 

## 5. Conclusions

We conclude that the storage carbohydrate glycogen, even though present in large amounts, does not have a major role in maintaining viability during long-term culture of *C. albicans*. Neither growth nor morphology nor metabolic flexibility are negatively affected by glycogen deficiency. The salient long-term survival defect of a *C. albicans efg1/efg1* mutant is thus rooted in factors other than glycogen deficiency.

## Figures and Tables

**Table 1:** *C. albicans* strains used in this study.

Strain/Collection	Genotype	Author
Gene Replacement and Conditional Expression (GRACE) CaSS1-based (2356 conditional mutants); Enriched for putatively essential genes	One allele deleted with *HIS3*, second allele under control of tetracycline-repressible promoter	[18]
Homan et al. Transcriptional Regulator Knockout collection (165 knockout mutants)	*LEU2* and *HIS1* deletion of target genes in SN152 background *arg4∆/arg4∆ leu2∆/leu2∆ his1∆/his1∆ URA3/ura3∆::imm434 IRO1/iro1∆::imm434* Collection includes wildtype control with *LEU2* and *HIS1* integration	[19]
Noble et al. Morphogenetic switching/pathogenicity collection (674 knockout mutants)	*LEU2* and *HIS1* deletion of target genes in SN152 background *arg4∆/arg4∆ leu2∆/leu2∆ his1∆/his1∆ URA3/ura3∆::imm434 IRO1/iro1∆::imm434* Collection includes wildtype control with *LEU2* and *HIS1* integration	[20]
SN152	*arg4∆/arg4∆ leu2∆/leu2∆ his1∆/his1∆ URA3/ura3∆::imm434 IRO1/iro1∆::imm434*	[21]
CaMS626	SN152 with *gsy1::ARG4*	This study
CaMS627	SN152 with *gsy1::ARG4/gsy1::HIS1*	This study
CAI4	*ura3::imm434/ura3::imm434*	[22]
HLC52 (*efg1*)	*ura3::imm434/ura3::imm434* *efg1::hisG/efg1::hisG::URA3::hisG*	[17]

**Table 2:** List of mutants with low glycogen content isolated in the library screen.

NAME/SYST ORF	Collection	Glycogen mg/mg (% of WT)
CaSS1/WT	GRACE	0.046 +/− 0.007
SN250/WT	H, N	0.048 +/− 0.01
PGK1/C6_00750C_A	GRACE	0.019 +/− 0.001 (42%)
C3_06700C_A	GRACE	0.016 +/− 0.0001 (35%)
COQ1/CR_00570W_A	GRACE	0.018 +/− 0.004 (39%)
C4_03410W_A	GRACE	0.022 +/− 0.009 (48%)
C4_00660W_A	GRACE	0.019 +/− 0 (41%)
MSU1/C2_08550C_A	GRACE	0.019 +/− 0.004 (41%)
C1_13150W_A	GRACE	0.02 +/− 0.002 (43%)
C6_02150C_A	GRACE	0.017 +/− 0.002 (37%)
MSW1/C5_02770W_A	GRACE	0.016 +/− 0.004 (35%)
TPS2/C1_03380W_A	GRACE	0.025 +/− 0.01 (54%)
GPM1/C2_03270W_A	GRACE	0.02 +/− 0.006 (44%)
C3_03100C_A	GRACE	0.016 +/− 0.0005 (35%)
WAL1/CR_09650W_A	GRACE	0.015 +/− 0.001 (33%)
C1_01140C_A	GRACE	0.006 +/− 0.002 (12%)
COQ5/C2_05470W_A	GRACE	0.023 +/− 0.003 (49%)
CR_01120C_A	GRACE	0.016 +/− 0.002 (35%)
C5_05230C_A	GRACE	0.011 +/− 0.001 (25%)
C4_06000W_A	GRACE	0.02 +/− 0.003 (43%)
C1_01010W_A	GRACE	0.023 +/− 0.005 (49%)
TSC11/CR_07580C_A	GRACE	0.033 +/− 0.001 (71%)
SSK1/C1_13930W_A	GRACE	0.02 +/− 0.016 (44%)
EFG1/CR_07890W_A	H	0.005 +/− 0.001 (11%)
COX4/C2_01620W_A	N	0.024 +/− 0.0004 (49%)
SNF4/C6_03920W_A	N	0.009 +/− 0.0025 (18%)
PSR1/C3_00570C_A	N	0.018 +/− 0.007 (36%)

GRACE = Gene replacement and conditional expression collection, H = Transcriptional regulator knockout collection by Homan et al. [19], N = Morphogenetic switching/pathogenicity collection by Noble et al. [20].
